# The Modes of Action of MARTX Toxin Effector Domains

**DOI:** 10.3390/toxins10120507

**Published:** 2018-12-02

**Authors:** Byoung Sik Kim

**Affiliations:** Department of Food Science and Engineering, ELTEC College of Engineering, Ewha Womans University, Seoul 03760, Korea; b.kim@ewha.ac.kr; Tel.: +82-2-3277-4031

**Keywords:** bacterial protein toxin, MARTX toxin, effector domain, host–microbe interaction, host–pathogen interaction

## Abstract

Many Gram-negative bacterial pathogens directly deliver numerous effector proteins from the bacterium to the host cell, thereby altering the target cell physiology. The already well-characterized effector delivery systems are type III, type IV, and type VI secretion systems. Multifunctional autoprocessing repeats-in-toxin (MARTX) toxins are another effector delivery platform employed by some genera of Gram-negative bacteria. These single polypeptide exotoxins possess up to five effector domains in a modular fashion in their central regions. Upon binding to the host cell plasma membrane, MARTX toxins form a pore using amino- and carboxyl-terminal repeat-containing arms and translocate the effector domains into the cells. Consequently, MARTX toxins affect the integrity of the host cells and often induce cell death. Thus, they have been characterized as crucial virulence factors of certain human pathogens. This review covers how each of the MARTX toxin effector domains exhibits cytopathic and/or cytotoxic activities in cells, with their structural features revealed recently. In addition, future directions for the comprehensive understanding of MARTX toxin-mediated pathogenesis are discussed.

## 1. Introduction

To be a successful pathogen, host-invading bacteria produce various virulence factors. Examples range from catabolic utilization systems for host-derived nutrients that enable the in vivo growth of the pathogen to a series of cytopathic or cytotoxic molecules that disturb the host defense systems [1,2,3,4]. Among the latter bacterial arsenal, effector proteins of type III, type IV, and type VI secretion systems have been intensively studied in the context of host–microbe interaction since the proteins are directly translocated from the pathogens to the host cells [5,6]. In the cells, these effector proteins carry out their specialized functions such as sequestering, scaffolding, or catalytically modifying the host target molecules that result in perturbations in the host cell structure and signaling. Conversely, bacterial pathogens secrete effector proteins to build a more desirable environment for their in vivo survival, adaptation, and proliferation [7].

A multifunctional autoprocessing repeats-in-toxin (MARTX) toxin was originally identified in a cholera agent *Vibrio cholerae* as an accessory cytotoxic toxin for which the gene is physically linked to the cholera toxin prophage [8]. Since then, genes encoding homolog proteins have been discovered in a range of other pathogenic bacteria, some of which have been characterized as important virulence factors [9,10,11]. In particular, it has been shown that the inactivation of the *rtxA1* gene encoding a MARTX toxin in *V. vulnificus* significantly attenuates the virulence of this life-threatening human and aquatic animal pathogen [12,13,14,15]. According to the Identical Protein Groups database from the National Center for Biotechnology Information (NCBI) (https://www.ncbi.nlm.nih.gov/ipg/, as of July 2018), MARTX toxins are distributed in the following 12 bacterial genera, Aeromonas, Aliivibrio, Chromobacterium, Moellerella, Moritella, Photobacterium, Photorhabdus, Proteus, Psychromonas, Vibrio, Xenorhabdus, and Yersinia.

**Figure 1:**
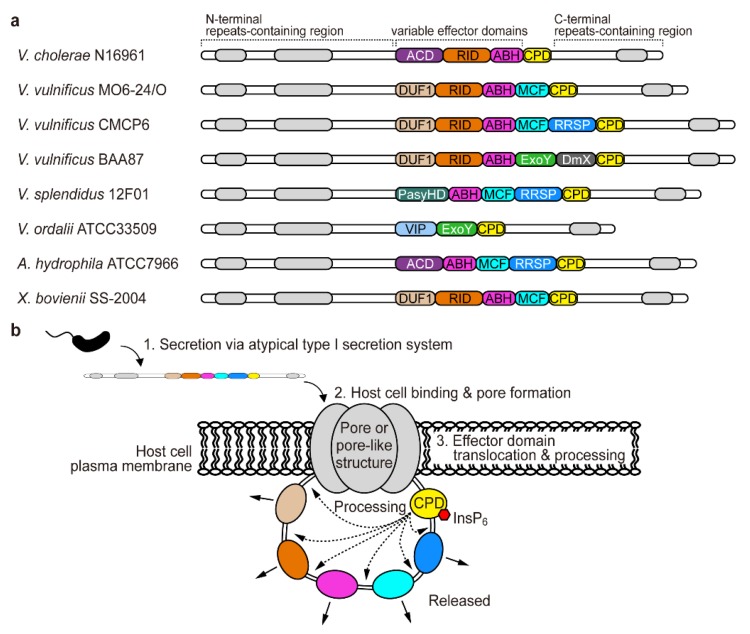
Multifunctional autoprocessing repeats-in-toxin (MARTX) toxin delivers various effector domains into the host cell cytosol. (**a**) Schematic diagrams of MARTX toxins from various pathogens. The sequences used in the analysis were downloaded from the NCBI website: *Vibrio cholerae* N16961 (WP_010895441.1); *V. vulnificus* MO6-24/O (WP_015728045.1); *V. vulnificus* CMCP6 (WP_011081430.1); *V. vulnificus* BAA87 (WP_039507922.1); *V. splendidus* 12F01 (WP_004732217.1); *V. ordalii* ATCC33509 (WP_010319615.1); *Aeromonas hydrophila* ATCC7966 (WP_011705266.1); and *Xenorhabdus bovienii* SS-2004 (WP_012987644.1). (**b**) The steps in MARTX toxin intoxication. After effector domain translocation, InsP_6_ activates the cysteine protease domain (CPD) which then autoprocesses the inter-effector domain regions to release each effector domain from the holo-MARTX toxin.

The feature of a MARTX toxin which discriminates it from other bacterial exotoxins is that this single polypeptide protein consists of multiple effector domains and arms containing repeated sequences (Figure 1a) [11,16]. Markedly, the effector domains vary among the MARTX toxins from different bacterial species or even from different strains in the same species, while the arms containing repeats are relatively conserved [17,18].

It has been shown that the MARTX toxins are secreted from the bacterium to the extracellular space through an atypical type I secretion system [19,20]. Once secreted, the MARTX toxin binds to the host cell plasma membrane and forms a pore or pore-like structure probably by using the amino- and carboxyl-terminal arms (Figure 1b) [14,21]. Although both molecular structure of the pore and precise mechanism of the translocation are remained to be elucidated, it has been thought that the MARTX toxin effector domains are delivered into the host cell cytosol through that pore (Figure 1b) [16,21,22]. In the host cell, inter effector domain regions are processed by another conserved domain of the toxin, a cysteine protease domain (CPD), and thus the effector domains are released from the holo-MARTX toxin (Figure 1b). Because the CPD forms an active structure only if it binds to the eukaryotic cell-specific molecule inositol hexakisphosphate (InsP_6_), the effector processing predominantly occurs in the host cell cytosol [23,24,25]. Subsequently, each of the released effector domains targets the host molecule(s) to subvert the normal physiology of the cells.

Details of the production, secretion, and CPD-mediated autoprocessing of MARTX toxins have already been reviewed in other recent articles [26,27,28,29]. Therefore, the main focus of this review is on the molecular mechanism of each MARTX toxin effector domain along with its predicted or recently solved three-dimensional structure. In addition, perspectives and future directions related to MARTX toxin-mediated pathogenesis, especially with the Vibrio infection cases, are discussed.

## 2. The Repertoire of the MARTX Toxin Effector Domains

Because of its original characterization in *V. cholerae* and critical role in the pathogenesis of *V. vulnificus*, MARTX toxins and their effector domains have mostly been studied in these strains. *V. cholerae* seventh-pandemic El Tor strain N16961 produces a MARTX toxin containing three effector domains: actin cross-linking domain (ACD), Rho GTPase-inactivation domain (RID), and alpha/beta hydrolase domain (ABH) (Figure 1a) [11]. Other El Tor and El Tor-like strains also encode MARTX toxins with the same effector domains as consistent with the fact that their whole genomes are quite stable [30]. Compared to this, different strains of *V. vulnificus* produce much diverse MARTX toxins [18] (Figure 1a). Clinical isolate MO6-24/O produces a MARTX toxin containing domain of unknown function at the first position (DUF1), RID, ABH, and makes caterpillars floppy-like domain (MCF). Other clinical isolates CMCP6 and YJ016 have these four domains as well as one additional domain, Ras/Rap1-specific endopeptidase (RRSP) [31]. Recently emerged biotype 3 strain BAA87 contains ExoY-like adenylate cyclase domain (ExoY) and domain X (DmX) instead of MCF and RRSP, respectively [32]. In addition to the previously mentioned effector domains, VIP2 (vegetative insecticidal protein 2 homology domain predicted to have ADP-ribosylation activity) and PasyHD (a domain of unknown function from the MARTX toxin of *Photorhabdus asymbiotica*) are other composite members of the MARTX toxin effector domains identified in the Vibrio species and other bacterial genera [27]. Of note, new effector domains should be found if more bacterial genomes are sequenced and carefully inspected.

## 3. Cytoskeleton Disruption by the Actin Cross-Linking Domain (ACD)

The first characterized MARTX toxin effector domain is an ACD from *V. cholerae*. During co-incubation with HEp-2 cells, the bacterial strain producing a functional MARTX toxin made cells rapidly round-up, covalently cross-linking all monomeric actins in the cytosol [33]. Following mutational and cell biological analyses revealed that a certain region of the MARTX toxin (amino acids 1963–2372 according to the sequence of MARTX toxin from *V. cholerae* N16961) is responsible for this cytoskeleton disruption, and thus the region was assigned as an ‘Actin Cross-linking Domain’ [34]. Intriguingly, a homolog of ACD had been found in the *V. cholerae* type VI secretion effector, VgrG-1, as an accompanying domain. Although the amino acid sequence identity between the ACD of the MARTX toxin and that of VgrG-1 is not very high (61%), both ACDs are able to cross-link cellular actins, which indicates that the domain is not only restricted in MARTX toxins [35].

**Figure 2:**
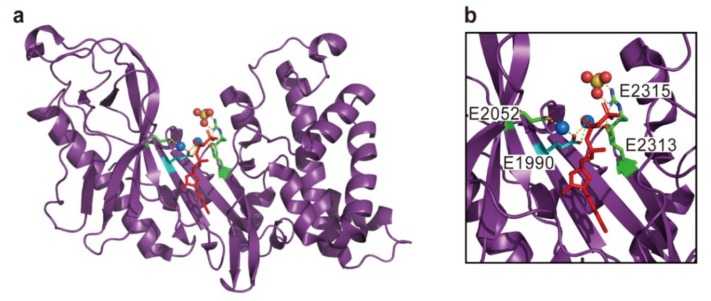
The crystal structure of actin cross-linking domain (ACD) in *V. cholerae* VgrG1 (Protein Data Bank (PDB) code 4DTH). Overall structure (**a**) and close-up view for active site (**b**) are shown. Critical and important residues for the actin cross-linking reaction are represented with cyan and green sticks, respectively. An adenosine triphosphate (ATP) molecule is shown with a red stick. A sulfate ion mimicking the position of Glu270 in actin and two Mg^2+^ ions are shown in the sphere model. The residues are numbered according to the sequence of multifunctional autoprocessing repeats-in-toxin (MARTX) toxin from *V. cholerae* N16961. All effector domain structures in this review were visualized using PyMOL software ver. 1.5.0.4 (Schrödinger, LLC, New York, NY, USA).

ACD forms an intermolecular isopeptide linkage between the Glu270 residue of a G-actin monomer and the Lys50 residue of the next one that results in the formation of nonfunctional actin oligomer chains [36]. In vitro experiments with recombinant ACD and actin proteins have demonstrated that adenosine triphosphate (ATP) and divalent cations such as Mg^2+^ are essential for the reaction [37]. Indeed, a structural analysis has revealed that ATP and two Mg^2+^ ions are tightly bound in the active site of VgrG-1 ACD (Figure 2) [38]. Notably, a few β-strands in the ACD active site harboring the critical Glu residue for Mg^2+^ coordination (E1990 in the case of MARTX toxin from *V. cholerae* N16961) are quite similar to those found in the glutamine ligase/γ-glutamylcysteine synthetase. Accordingly, the following two-step mechanism for ACD-mediated actin cross-linking was proposed. Briefly, the γ-phosphate of an ATP molecule is transferred to the carboxylic group of the Glu270 side chain of one actin molecule. The neighboring cations catalyze this transfer and stabilize the transferred phosphate group in the intermediate molecule. Subsequently, the ε-amino group of the Lys50 of the next actin molecule executes a nucleophilic attack, thereby expelling the transferred phosphate and forming an isopeptide bond [38,39].

Until recently, the cytotoxicity of ACD was believed to be the results of gradual reduction of the G-actin pool and the consequent depolymerization of the F-actin filaments in the intoxicated cells [36]. However, Heisler et al. have doubted this and hypothesized that the ACD would initiate a toxicity cascade by producing cross-linked actin species, which may act as second messengers [40]. Certainly, they found that the nonfunctional actin oligomers produced by ACD are actually highly toxic to the cells as they tightly bind to and efficiently inhibit major actin assembly proteins such as formin, Ena/vasodilator-stimulated phosphoprotein (VASP), Spire, and Arp2/3 complex [40,41]. Therefore, ACD proficiently disrupts the cell cytoskeleton, inhibits the engulfing activity of phagocytic immune cells, and consequently prevents the clearance of the invading bacteria from the host [22].

## 4. Cell Rounding by the Rho GTPase-Inactivation Domain (RID)

In contrast to ACD, which directly targets actin molecules, RID indirectly depolymerizes actin filaments by inactivating Rho-family GTPases. During the characterization of ACD, Sheahan and Satchell had noticed that the *V. cholerae* mutant strain expressing the ΔACD MARTX toxin (ACD-domain deleted MARTX toxin) maintains the MARTX toxin-dependent cell-rounding activity, although the cell rounding occurs slowly. In parallel, they had carried out infection experiments with *V. vulnificus* and observed similar cell rounding [42]. Notably, the mutant *V. cholerae* strain and the used *V. vulnificus* strain do not exhibit the actin cross-linking activity. Therefore, the results suggest that a certain region presenting in both the *V. cholerae* and *V. vulnificus* MARTX toxins can induce the host cell rounding in an ACD-independent manner. Among such regions, a domain corresponding to amino acids 2552–3099 (according to the sequence of MARTX toxin from *V. cholerae* N16961) actually caused host cell rounding when ectopically overexpressed [42]. Interestingly, if delivered into the cells by *V. cholerae*-mediated intoxication or anthrax toxin-mediated endocytosis (the LF_N_/PA system) [37,43], this domain affected the cellular levels of GTP-bound active RhoA, a key small GTPase regulating cell cytoskeleton, but not the total RhoA. Cellular levels of active Rac1 and Cdc42 were similarly affected, and thus the domain has been named as the ’Rho GTPase-Inactivation Domain’ [42].

**Figure 3:**
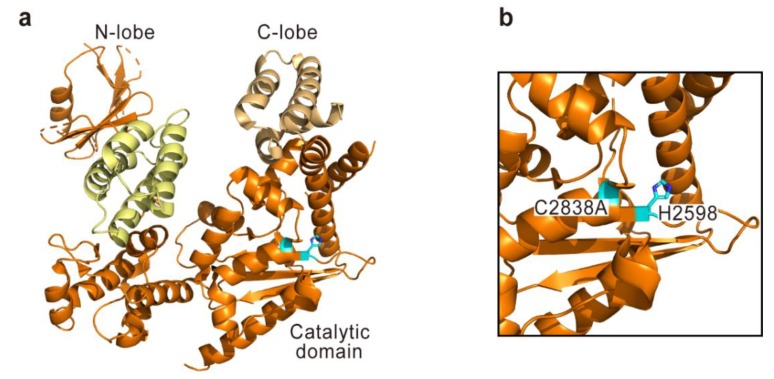
The crystal structure of Rho GTPase-inactivation domain (RID) in the *V. vulnificus* MARTX toxin (PDB code 5XN7). Overall structure (**a**) and close-up view for active site (**b**) are shown. Catalytic residues are represented with cyan sticks. A membrane localization domain (MLD) and a four-helix pair are represented in light yellow and light orange, respectively. The residues are numbered according to the sequence of MARTX toxin from *V. vulnificus* MO6-24/O.

Recently, the three-dimensional structure and molecular mechanism of *V. vulnificus* RID have been disclosed [44]. Overall, RID forms a twisted U-shape structure consisting of an N-terminal lobe (N-lobe) and a C-terminal lobe (C-lobe) (Figure 3). In the N-lobe, a membrane localization domain (MLD) that brings the entire RID to the host cell plasma membrane by interacting with anionic lipids is present [45]. Certainly, the N-lobe has shown a specific interaction with phosphatidylinositol 4,5-bisphosphate in a PIP (phosphatidylinositol phosphate)-strip overlay experiment [44]. In the case of the C-lobe, a catalytic domain and a four-helix pair, the function of which has not yet been elucidated, are present. Importantly, the catalytic domain contains critical residues (Cys2838 and His2598 according to the sequence of MARTX toxin from *V. vulnificus* MO6-24/O) for the cytopathic function of RID [46]. When structural homologs of the RID catalytic core were searched for in the Dali server, a human fatty acid acyltransferase HRAS-like tumor suppressor 3 (HRASLS3) protein was identified, thus enabling the prediction of the RID-catalyzed biochemical reaction. By adopting a series of biochemical and mass spectrometric analyses, Zhu’s group successfully revealed that RID is an N^ε^-fatty acyltransferase transferring long-chain (more than 10 carbons) fatty acid from acyl-CoA to Lys residues in the C-terminal polybasic region of Rho-family GTPase proteins [44]. Through fatty acylation, RID forces Rho proteins to become anchored to a plasma membrane, thereby disrupting the functional cycling of Rho-family GTPases from the membrane to the cytosol and vice versa. Moreover, this lipidation affects interactions between Rho-family GTPases and their cognate guanine nucleotide exchange factors (GEFs) such as Tiam1 and DOCK2. This further prevents the activation of Rho-family GTPases and the following interactions with downstream effector proteins such as PAK1. Disruptions in these Rho-mediated signaling pathways eventually affect the cell cytoskeleton, the integrity of the epithelial cell monolayer, the production of reactive oxygen species, the migration of infected cells, and phagocytosis [22,44].

The *Shigella flexneri* type III secretion effector IcsB has recently been shown to carry out the same fatty acylation activity, although the sequence identity between the catalytic domain of RID and that of IcsB is very low (12–16%) [46,47]. Interestingly, IcsB modifies not only the Rho-family proteins but also other non-GTPase substrates like SNARE proteins. Nonetheless, the results indicate that RID and IcsB are founding members of the long-chain fatty acyltransferases showing an unprecedented cytopathic mechanism in host–microbe interaction [47].

## 5. Inhibition of Autophagic/Endosomal Trafficking by the Alpha/Beta Hydrolase Domain (ABH)

**Figure 4:**
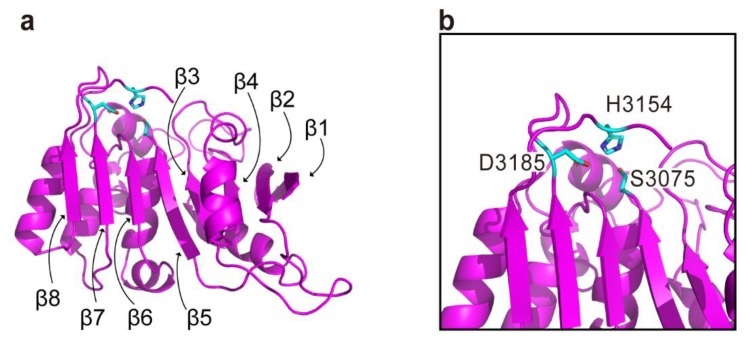
The modeled structure of alpha/beta hydrolase domain (ABH) generated by using the structure of *P. haloplanktis* FGH (PDB code 3LS2) as a template. Overall structure (**a**) and close-up view for active site (**b**) are shown. Catalytic residues are represented with cyan sticks, and eight beta-strands in the typical alpha/beta hydrolase fold are indicated. The residues are numbered according to the sequence of MARTX toxin from *V. vulnificus* MO6-24/O.

The CPD-mediated in vitro autoprocessing of the *V. cholerae* MARTX toxin had revealed another distinct domain other than ACD and RID. Since the typical folding of alpha/beta hydrolase was deduced from the amino acid sequence of this domain, the latter has been named as the ‘Alpha/Beta Hydrolase’ domain [11,24]. No three-dimensional structure of ABH from any of the MARTX toxins has been determined, but a sequence-based structural homolog search for the *V. vulnificus* ABH via the HHpred server [48] indeed reveals various hydrolases as the most probable structural homologs. These include EstA (an esterase protein from *Streptococcus pneumoniae*) and FGH (an S-formylglutathione hydrolase from *Pseudoalteromonas haloplanktis*) [49,50]. In the structural model of ABH generated via the SWISS-MODEL server [51] using *P. haloplanktis* FGH as a template, a typical alpha/beta hydrolase topology consisting of an eight-stranded β-sheet and surrounding α-helices is clearly shown (Figure 4). Furthermore, the previously characterized residues forming the catalytic triad Ser3075, His3154, and Asp3185 (according to the sequence of MARTX toxin from *V. vulnificus* MO6-24/O) are markedly present in a putative catalytic site [52], suggesting lipase, esterase, or other hydrolase activities of ABH.

In contrast to ACD and RID, ABH had not exhibited any distinctive cytopathic or cytotoxic phenotypes in transfection-based analyses, and thus its molecular function had remained elusive for around a decade. However, the *V. cholerae*-mediated natural delivery of the ABH suggested that the domain might affects host cell signaling. This is because the solely delivered ABH significantly activated the host small GTPase Cdc42 in a catalytic His residue-dependent manner [22].

More recently, the Satchell group found that ABH is a phosphatidylinositol 3-phosphate (PtdIns3P)-specific phospholipase A1 [52]. Certainly, the recombinant ABH protein specifically bound to PtdIns3P in the PIP-strip overlay experiment. In addition, mass spectrometry analyses of the in vitro reaction products clearly demonstrated that this lipase/esterase cleaves off a fatty acid on the *sn1* position of glycerol phosphate in the PtdIns3P molecule. Since PtdIns3P is a crucial molecule for autophagosome formation and endolysosomal pathways [53,54], the authors further examined whether ABH affects these two fundamental cellular processes. As expected, both transiently overexpressed and naturally delivered ABHs reduced the intracellular PtdIns3P levels and blocked autophagic/endosomal pathways [52]. This molecular function may also explain the ABH-mediated Cdc42 activation because the reduced level of PtdIns3P would eventually affect the cellular levels of other phosphoinositides, which actually modulate the activity of various Rho-family GTPases including Cdc42 [55].

## 6. Upregulation of Prohibitin Expression by the Domain of Unknown Function in the First Position (DUF1)

DUF1 is a domain at the first position in the effector region of the MARTX toxin from the *V. vulnificus* MO6-24/O, CMCP6, or BAA87 strains. Unlike with other MARTX effector domains, neither the solved three-dimensional structure nor the predicted model of DUF1 is available at this time. Importantly, no certain catalytic residues have been assigned or even assumed, and therefore it is still unknown if this domain functions as an enzyme or as a scaffolding protein. Although not exactly matched with DUF1, the ’RtxA1-D2’ region containing both DUF1 and the amino-terminal part of RID has been studied, which has given some insights into the function of DUF1 [56]. When ectopically overexpressed, RtxA1-D2 made HeLa cells balloon, which is reminiscent of an early pathomorphology of *V. vulnificus* infection [57]. To find out the host cell partners of this cytotoxic domain, yeast two-hybrid screening was adopted and the prohibitin 1 (PHB1) protein was identified as a putative partner. Indeed, pretreatment of anti-PHB1 antibodies or a small interfering RNA-mediated knockdown of PHB1 attenuated the cytotoxicity of RtxA1-D2 against HeLa cells. Furthermore, the expression of PHB1 was markedly increased when the cells were treated with the MARTX toxin secreting *V. vulnificus* but not a MARTX toxin deficient one. Collectively, PHB1 has been assumed as an initial receptor protein for the early binding of the MARTX toxins and to complete a positive feedback route for the further bindings [56]. However, many MARTX toxins from other pathogens notably lack a DUF1 domain [11], indicating that this assumption should be carefully reexamined with an exact molecular mechanism of this not-as-yet characterized domain.

## 7. Apoptotic Cell Death Caused by the Makes Caterpillars Floppy-Like Domain (MCF)

An early study investigating the functions of the *V. vulnificus* MARTX toxin had revealed that it induces the apoptotic death of host cells [58]. Specifically, the cellular levels of cleaved caspase-3 (one of the executioner caspases) and inactivated poly-(ADP-ribose) polymerase (PARP-γ; a DNA repair protein) were significantly increased when the wild-type *V. vulnificus* but not a MARTX toxin-deficient nor a secretion defective mutant strain was treated onto the human epithelial cells. Consistent with this, cytochrome *c* was released from the mitochondria in the case of the wild-type strain treatment. However, the MARTX toxin effector domain responsible for this apoptotic cell death has only been investigated recently and was suggested as an MCF domain [59].

The domain name MCF stands for ‘Makes Caterpillars Floppy-like’ since the amino acid sequence of MCF domain shows a significant similarity to that of insecticidal toxins Mcf1 and Mcf2 from *Photorhabdus luminescens* [11,16]. When injected into the host, Mcf1 actually induced a floppy phenotype in insect larvae [60]. Although similar phenotypes have never been found for the MCF domain of a MARTX toxin, its biochemical characteristics and intoxication consequences have been intensely investigated [59,61].

**Figure 5:**
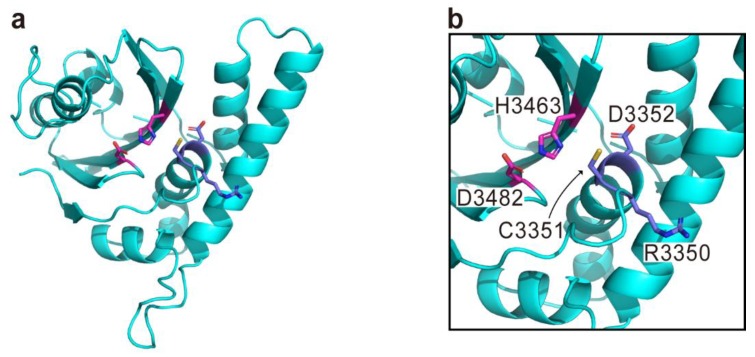
The modeled structure of makes caterpillars floppy-like domain (MCF) generated by using the structure of *P. syringae* AvrPphB (PDB code 1UKF) as a template. Overall structure (**a**) and close-up view for active site (**b**) are shown. Although MCF is predicted to have a typical catalytic triad (Cys3351, His3463, and D3482) for the C58 cysteine peptidase family, the previously proposed catalytic tripeptides are Arg3350, Cys3351, and D3352 represented with violet sticks [61]. The residues are numbered according to the sequence of MARTX toxin from *V. vulnificus* MO6-24/O.

When ectopically overexpressed in HeLa cells, the MCF domain showed amino-terminal autoprocessing. Indeed, such an event was not observed if a putative catalytic tripeptide residue (either R3350, C3351, or D3352 according to the sequence of MARTX toxin from *V. vulnificus* MO6-24/O) was substituted for Ala. In addition, the same catalytic residues were found to be critical for the MCF-mediated cell shrinking [61]. Although MCF is predicted to have a typical C58 cysteine peptidase fold similar to the *Pseudomonas syringae* type III secretion effector AvrPphB [62] (Figure 5), the above mutational experimental results have placed the MCF domain and its homologs at the unprecedented subgroup of the C58 peptidase containing consecutive tripeptides as catalytic residues [61]. In vitro experiments with a recombinant MCF protein further showed that the autoprocessing is induced by a certain host factor(s) which is heat-stable, small, and proteinaceous [61].

The followed cell biological and bacterial challenge studies have revealed the consequence of MCF intoxication [59]. After autoprocessing, the MCF domain induced depolarization of the mitochondrial membrane and prompted the release of cytochrome *c*. Meanwhile, pro-apoptotic factors Bax and Bak were upregulated along with the activation of caspases 3, 7, and 9. Collectively, the MCF-intoxicated cells underwent programmed apoptotic cell death. However, the shrinking phenotype of MCF intoxication notably persisted even in the presence of pan-caspase inhibitor, suggesting that MCF does not directly induce the caspase-mediated apoptosis [59]. Instead, MCF may affect another cellular molecule(s) the cleavage of which indirectly provokes apoptotic cell death. Such authentic MCF-target protein(s) has so far remained undiscovered. Because another recent report from an independent group has also pointed out MARTX toxin-mediated mitochondrial dysfunction [57], such target protein(s) probably has a role in maintaining the integrity of subcellular organelles.

## 8. Dysregulation of Host Cell Signaling by the Ras/Rap1-Specific Endopeptidase (RRSP)

Before the discovery of its molecular targets in host cells, the RRSP domain had been called DUF5 meaning ‘domain of unknown function in the fifth position’ of the MARTX toxin from *V. vulnificus* CMCP6 [17]. Intriguingly, *V. vulnificus* strains with the toxin variants omitting this domain were less virulent to mice, indicating a significant role of DUF5 in the pathogenesis of invading bacteria [31,63]. Bioinformatic and structural modeling analyses suggested that this domain may belong to the Tiki superfamily, and it has a similar structure to certain parts (C1 and C2 subdomains) of the *Pasteurella multocida* toxin (PMT) [64,65,66]. Indeed, a series of molecular functional studies revealed that the ectopic expression of DUF5 or the direct delivery of recombinant DUF5 via the LF_N_/PA system is cytotoxic to host cells [64,67].

One of the most important findings from previous studies is that DUF5 directly processes a peptide bond between Tyr32 and Asp33 in the switch I region of the cellular Ras and Rap1 proteins, and reflecting this activity, DUF5 has been renamed as the ’Ras/Rap1-Specific endoPeptidase’ domain [67]. Notably, these two small GTPases are crucial for host innate immune defense systems as well as cell proliferation [68,69]. Thus, the clear characterization of RRSP would give new opportunities to treat not only RRSP-mediated bacterial infectious diseases but also various malignancies related to Ras mutation [70]. Accordingly, the molecular basis for the Ras/Rap1 recognition by RRSP has been further examined, and biochemical analyses using switch I region-swapped chimeric GTPases have revealed that the entire switch I region of Ras/Rap1 but not their guanine nucleotide binding state is critical for the recognition [71].

**Figure 6:**
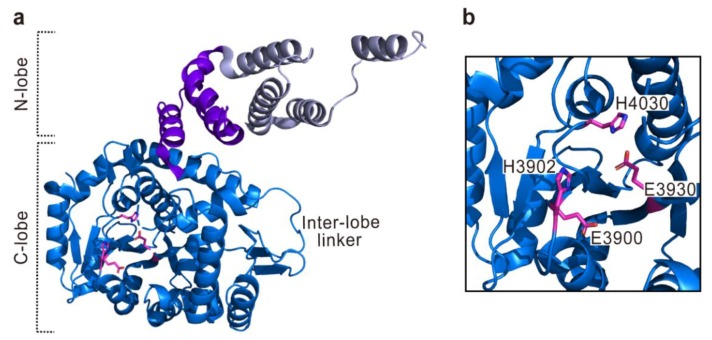
The crystal structure of Ras/Rap1-specific endopeptidase (RRSP) in *V. vulnificus* MARTX toxin (PDB code 6A8J). Overall structure (**a**) and close-up view for active site (**b**) are shown. The 2His/2Glu active site residues are represented with magenta sticks. The membrane localization domain (MLD) and three additional helices in the N-lobe are represented in light blue and violet, respectively. The residues are numbered according to the sequence of MARTX toxin from *V. vulnificus* CMCP6.

Very recently, two independent groups have solved the three-dimensional crystal structure of RRSP [72,73]. Indeed, the overall structure of RRSP is almost the same as that of the C1/C2 domains of PMT (RMSD = 2.79 Å for 404 C_α_ atoms), although their amino acid sequences are largely different (identity, 26%) [73]. Specifically, RRSP consists of N- and C-lobes connected by an interlobe linker, which instills flexibility between the lobes (Figure 6). In the N-lobe, an MLD domain comprising four helices is located followed by three additional helices. Similar to that in RID, the MLD domain brings entire RRSP to the plasma membrane of the intoxicated host cells [73]. In the C-lobe of RRSP, a typical Tiki peptidase fold on which a 2His/2Glu active site (composed of His3902, His4030, Glu3900, and Glu3930 according to the sequence of MARTX toxin from *V. vulnificus* CMCP6) is located (Figure 6). However, the C-lobe alone was not able to process the Ras protein in vitro, suggesting that the N-lobe while binding to the membrane may alter the conformation of C-lobe to be more suitable for Ras/Rap1 recruitment [73]. Importantly, the Ras protein only showed a local disorder at the switch I region and its overall tertiary structure remained even after RRSP-mediated cleavage. Despite this, the resulting Ras protein was unable to interact with its GEF protein Son of Sevenless (SOS) or with the downstream effector protein Raf, and thus the mitogen-activated protein kinase pathway in the intoxicated cell was significantly dysregulated [67,72]. If one solved the crystal structure of the RRSP-Ras complex, a precise molecular mechanism of this Tiki family endopeptidase could be further elucidated.

## 9. Intracellular Accumulation of Cyclic AMPs by the ExoY-Like Domain (ExoY)

In the amino acid sequence analyses, one of two newly found effector domains in the MARTX toxin of *V. vulnificus* biotype 3 strain showed a sequence identity of 19 to 20% with *Bordetella pertussis* adenylate cyclase toxin CyaA or *Bacillus anthracis* edema factor (EF). If the sequence was compared with that of the *P. aeruginosa* type III secretion effector ExoY (PaExoY), the identity was even higher (26%) and thus the effector domain has been named as the ‘ExoY-like’ domain (ExoY) [32]. Indeed, the catalytic regions (CR1, CR2, and CR3) containing essential residues for CyaA, EF, or PaExoY are well conserved in ExoY [74,75]. Moreover, when Chinese hamster ovary cells were infected with the ExoY-positive *V. vulnificus*, intracellular level of cyclic adenosine monophosphate (cAMP) was significantly increased. Consistent with this, the recombinant ExoY protein was able to convert ATPs to cAMPs in vitro, although the reaction was somewhat inefficient [32].

Since the substrate nucleotides are commonly present in both eukaryotic and prokaryotic cells, CyaA and EF have evolved to become activated only if the proteins interact with calmodulin (CaM), a calcium-binding protein found in eukaryotic cells [76,77]. However, neither PaExoY nor ExoY is activated by CaM, and the identity of the activating factor remained uncharacterized until Mechold’s group disclosed that it was an actin [78]. They indeed discovered that filamentous F-actins rather than monomeric G-actins induce the nucleotide cyclase activity of PaExoY. Similarly, the in vitro activity of the ExoY domain from *V. nigripulchritudo* MARTX toxin increased more than 10,000-fold in the presence of actin molecules, although G-actins in this case [78,79]. Unlike with PaExoY, which can produce not only cAMP but also cGMP, cUMP, and cCMP, the ExoY domains from Vibrio MARTX toxins exhibited only an adenylate cyclase activity [32,78]. Therefore it seems like that much narrower and more specific cell signaling pathways such as a pro-inflammatory cytokine production pathway would be affected by them [80].

**Figure 7:**
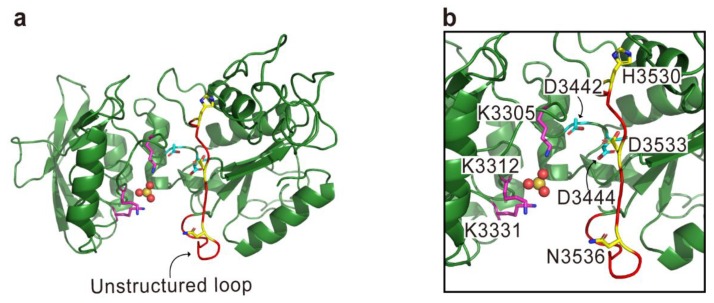
The modeled structure of ExoY generated by using the structure of *P. aeruginosa* type III secretion effector ExoY (PaExoY) (PDB code 5XNW) as a template. Overall structure (**a**) and close-up view for active site (**b**) are shown. The structure was analyzed based on the analogy of the CyaA and Edema factor (EF) structures [76,77]. ATP-binding essential residues on the critical regions (CR1, CR2, and CR3) are represented with magenta, cyan, and yellow sticks, respectively. A sulfate ion mimicking the phosphate group of ATP is shown in the sphere model. The residues are numbered according to the sequence of MARTX toxin from *V. vulnificus* BAA87.

Recently, the three-dimensional structure of PaExoY was solved at a resolution of 2.2 Å via in situ proteolysis-assisted crystallization [75]. Figure 7 shows a structural model of the *V. vulnificus* ExoY domain generated by using the structure of PaExoY as a template. Similar to PaExoY, CyaA, or EF, the ExoY is likely to consist of two subdomains with a catalytic site between them. The three conserved CRs involving substrate binding and catalysis are indeed gathered near the site (Figure 7). Importantly, K3305, K3312, and K3331 (according to the sequence of MARTX toxin from *V. vulnificus* BAA87), which are the residues predicted to hold the α-phosphate of substrate ATP, and D3442 and D3444, which are the residues predicted to stabilize the β-and γ-phosphates of ATP via Mg^2+^ ions, are completely conserved in the CR1 and CR2 regions of ExoY, respectively. The residues predicted to bind to the adenine moiety of ATP, namely H3530, D3533, and N3536, are on the unstructured loop of the CR3 region (Figure 7). This unstructured region will probably be stabilized and form a certain structure upon the binding of actin, as the corresponding ‘switch B’ region of EF does during CaM binding [76].

## 10. Golgi Disruption by the Domain X (DmX)

**Figure 8:**
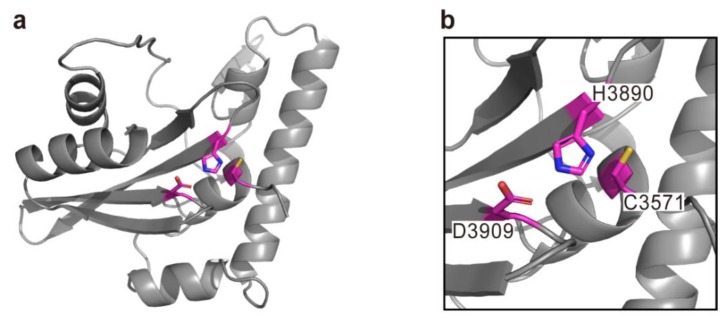
The modeled structure of DmX generated by using the structure of *P. syringae* AvrPphB (PDB code 1UKF) as a template. Overall structure (**a**) and close-up view for active site (**b**) are shown. The predicted catalytic triad is represented with magenta sticks. The residues are numbered according to the sequence of MARTX toxin from *V. vulnificus* BAA87.

Another recently found effector domain in the MARTX toxin of the *V. vulnificus* biotype 3 strain is Domain X (DmX), which stands for the domain of no known function [32]. Because this domain shows no sequence similarity to any other previously characterized proteins, its function was initially deduced from the modeled structure. The HHpred server [48] predicted that the central region of DmX (amino acids 3748–3930 according to the sequence of MARTX toxin from *V. vulnificus* BAA87) may have a structure similar to the *P. syringae* AvrPphB, a cysteine peptidase effector protein (Figure 8) [62,81]. Analysis of the modeled structure has revealed potential catalytic triad residues C3571, H3890, and D3909, which are actually conserved in all of the DmX domains from various MARTX toxins. Indeed, when ectopically expressed in HEK 293T cells, the DmX showed catalytic triad-dependent N-terminal autoprocessing and cell shrinkage. Further biochemical and cell biological analyses disclosed that the host ADP-ribosylation factor (ARF) protein directly binds to and induces the DmX autoprocessing, but only when the ARF is in a GTP-bound active state. Notably, the expressed DmX became localized to the Golgi, then disrupted the organelle structure and impeded general protein secretory pathways [81]. However, the cellular target protein directly affected by the peptidase activity of DmX still remains undisclosed.

## 11. Perspectives and Future Directions

This review covers the recent discoveries related to the structure and function of MARTX toxin effector domains. Although the biochemical mechanisms and direct target molecules for certain effector domains have evidently been characterized, those of DUF1, MCF, DmX, VIP2, and PasyHD still require further study. Moreover, since the MARTX toxin translocates a set of effector domains altogether at a defined stoichiometry, not only the function of each effector domain but also the integrated outcomes of the entire effector domain repertoire must be investigated [26].

In the meantime, the immunological consequences of effector domain functions should be considered. Indeed, MARTX toxins from *V. cholerae* and *V. vulnificus* have been shown to induce NLRP3-dependent caspase-1 activation followed by IL-1β secretion and pyroptosis [82]. A genome-wide analyses in European eels and laboratory mice infected with *V. vulnificus* also revealed that a number of immune-related genes like *clec1* (encoding C-type lectin 1), *il1r2* (interleukin-1 receptor type 2 precursor), and *cxcr4* (CXC chemokine receptor type 4) are differentially expressed in a MARTX toxin-dependent way [83,84]. However, the effector domains responsible for these immune responses have not yet been fully characterized. Although the RID domain has been reported not to induce Pyrin-dependent caspase-1 activation [85,86], fatty acylated Rac1 may prompt similar kinds of effector-triggered immunity responses. Likewise, the dysregulation of lipid metabolism by ABH or Golgi disruption by DmX could stimulate cellular innate sensors for immune surveillance. The MARTX toxin arms containing repeats might also contribute to such immune responses by acting similar to other pore-forming toxins [14,21,87].

The potency of MARTX toxins in the pathogenesis of bacteria is another subject for future study. In contrast to the *V. vulnificus* MARTX toxin that functions as the pathogen’s primary virulence factor [12,13,14,88], *V. cholerae* MARTX toxin is considered as an accessory toxin helping colonization [89]. One might guess that a MARTX toxin with more effector domains (e.g., *V. vulnificus* MARTX toxin) is much more virulent than one with fewer effector domains (e.g., *V. cholerae* MARTX toxin). However, this is not always true since a mutant *V. vulnificus* strain secreting ΔDUF1 or ΔABH MARTX toxin actually showed an increased virulence in mice [90]. This suggests that not simply the number of but also sorts of effector domains and their combination are important for toxin potency. In addition, small differences in the regions containing well-conserved repeats might affect the toxin potency. Certainly, an ‘effector-free’ variant of *V. vulnificus* MARTX toxin eventually lyses the host cells, while the same variant of *V. cholerae* MARTX toxin does not [21,22]. Otherwise, a production/secretion level of the toxin may be different among the pathogens, and this possibility could be examined by measuring the amount of produced MARTX toxin during infection.

Last, the molecular mechanisms of pore formation and effector domain translocation need to be clearly uncovered for a comprehensive understanding of MARTX toxins. Do they require specific molecule(s) on the host cell plasma membrane for the cell binding and/or pore formation? Which portion of the repeats-containing region is necessary for that binding and pore formation? Can we use antibodies targeting that portion as prophylactic/therapeutic agents against MARTX toxin intoxication? How can we capture the structure of MARTX toxin translocons for a cryo-electron microscopic analysis? With the answers to these questions, we could expand our knowledge of toxin-mediated host–pathogen interactions and might be able to overcome MARTX toxin-triggered bacterial pathogenesis.
